# miRNA-29b Inhibits Prostate Tumor Growth and Induces Apoptosis by Increasing Bim Expression

**DOI:** 10.3390/cells8111455

**Published:** 2019-11-18

**Authors:** Subhayan Sur, Robert Steele, Xingyi Shi, Ratna B. Ray

**Affiliations:** Department of Pathology, Saint Louis University1100 South Grand Boulevard, St. Louis, MO 63104, USA; Subhayan.sur@health.slu.edu (S.S.); Robert.steele@health.slu.edu (R.S.); xingyi.shi@health.slu.edu (X.S.)

**Keywords:** miRNA-29b, prostate cancer, Bim, cytochrome C, PARP

## Abstract

Prostate cancer is one of the most common cancers among men. Currently available therapies improve patient survival against local prostate cancer but have shown severe side effects. Advanced prostate cancer is still incurable. Studies have suggested the involvement of non-coding RNAs, especially micro-RNAs (miRNAs), in the regulation of multiple cellular events in cancer and thus several clinical trials are ongoing using miRNAs mimics or inhibitors. We previously demonstrated that miRNA-29b-3p (miR-29b) was downregulated in prostate cancer and that the overexpression of miR-29b limited prostate cancer metastasis. However, the therapeutic potential of the miR-29b against prostate cancer remains unknown. Here, we evaluated the therapeutic role of miR-29b in in vivo prostate tumors in a mouse model. Intratumoral injection of mimic miR-29b significantly inhibited prostate cancer xenograft tumor growth in nude mice. Subsequent study demonstrated that the overexpression of miR-29b reduced prostate cancer cell PC3 proliferation in a time dependent manner and induced cell death. Mechanistic study using a cancer pathway specific transcriptomic array revealed a significant overexpression of the pro-apoptotic gene BCL2L11 (Bim) in the miR-29b overexpressed PC3 cells, which was further verified in PC3 cells overexpressing miR-29b. We also observed a significant induction of Bim protein in miR-29b treated xenograft tumors. The induction of cytosolic accumulation of cytochrome C and PARP cleavage in miR-29b overexpressed PC3 cells was observed. Thus, our results suggest that miR-29b can be used as a potential molecule for prostate cancer therapy.

## 1. Introduction

Prostate cancer is one of the most common cancers in men and the estimated incidence rate is 174,650 with 31,620 deaths in United States in 2019 [1]. The incidence rate and mortality is around 20-fold higher in developed countries due to diet and life style factors [2]. Though the current therapies for local prostate cancer, including surgical prostatectomy, chemotherapy, radiation therapy, immune therapy, and androgen deprivation therapy (ADT) improve survival rate, severe side effects remain. On the other hand, advanced prostate cancer remains an incurable disease. Therefore, there is an unmet need for developing additional therapy against prostate cancer.

MicroRNAs (miRNAs) are small endogenous non-coding molecules, that are 19–24 nt in length, regulate gene expression by degrading messenger RNAs (mRNAs), repressing protein synthesis or interacting with long non coding RNAs [3,4]. Each miRNA targets a wide range of molecules resulting in the alteration of cellular processes. Deregulation of miRNAs was seen in cancers [3,5]. Further, miRNAs play important roles as potential diagnostic and prognostic markers in several cancers [3,4,6]. Different pre-clinical studies also showed that suppression of oncogenic miRNAs or induction of tumor suppressor miRNAs either alone or in combination with chemotherapeutic drugs inhibit cell proliferation, migration, invasion and induce apoptosis and drug sensitivity [3,7]. Locked nucleic acid (LNA)-modified antisense oligonucleotide of oncogenic miRNAs and mimic of tumor suppressor miRNAs showed potential roles in Phase 1 and 2 clinical trials [6]. Phase 3 and 4 clinical trials with the candidate miRNAs are about to begin [6]. Alteration in a number of miRNAs expression was evident in miRNA profile analysis and data base dependent meta-analysis in prostate tumors compared to adjacent non-tumor tissues, and in early relapse samples compared to non-relapse cancer patient samples [8,9]. We first demonstrated that miRNA-29b (miR-29b) expression is significantly lower in prostate cancer and inhibits prostate cancer metastasis [10,11]. Lower expression of miR29b in prostate cancer patients and its association with metastasis was also reported recently [12]. Further, the c-Myc proto-oncogene was found to repress miR-29b transcription, and upregulation of c-Myc promoter binding protein (MBP-1) could enhance miR-29b expression in prostate cancer [11,13,14]. Other studies showed downregulation of miR-29b in different cancers, and miR-29b targets several genes of cell survival, angiogenesis, metastasis, fibrosis, and epigenetic modification pathways [15,16]. The therapeutic potential of miR-29b against prostate cancer is not being examined. In this study, we evaluated the therapeutic potential of miR-29b in a pre-clinical model. We developed prostate xenograft tumor in nude mice implanting PC3 cells and miR-29b was delivered intratumorally. We observed regression of tumor growth and induction of apoptotic cell death.

## 2. Methods

### 2.1. Cell Culture and Mimic Transfection

Human prostate cancer cell lines PC3 and DU145, were procured from American Type Culture Collection and maintained in Dulbecco’s Modified Eagle’s Medium containing 10% FBS, 100 U/mL penicillin, and 100 μg/mL streptomycin in a humidified CO_2_ incubator. Cells (2.5 × 10^5^) were plated using 6-well plates overnight. Cells were transfected with 50 nM of control or mimic for miR-29b-3p (Assay ID: MC10103, ThermoFisher Scientific) mixed with Opti-MEM and Lipofectamine RNAiMAX (Invitrogen) according to manufacturer’s protocol. All the analysis was performed in triplicate.

### 2.2. In Vivo Studies

Animal experiments were performed according to the NIH guidelines, following a protocol approved by the Institutional Animal Care and Use Committee of Saint Louis University. Nude mice (6-week-old males) were purchased from Charles River Laboratories, and housed in a specific pathogen-free animal facility at the Saint Louis University. PC3 cells were resuspended in 100 μL serum-free medium, mixed with 40% Matrigel (BD Biosciences) and injected (2 × 10^6^/site) subcutaneously into hind flank of each mouse (*n* = 20). When the average tumor volumes reached 70 mm^3^, tumor bearing mice were randomly divided into two groups, control and experimental. Then, 10 µg of mimic miR-29b or control oligo complexed with siPORTamine (Invitrogen) in 50 µL Opti-MEM was injected intratumorally at an interval of 4 days a total of seven times. Doses of miRNA was determined from our previous experiences. Tumor volume was measured using digital caliper twice a week and calculated using the formula *L* × *W*^2^ × 0.5 (*L*, length; *W*, width; all parameters in millimeters). All mice were sacrificed 5 weeks post PC3 cell implantation. After sacrificing, a portion of the tumor was snap-frozen and stored at −80 °C for biochemical analysis.

### 2.3. RNA Isolation and Quantitative Real-Time Reverse Transcriptase PCR

PC3 cells were transiently transfected with mimic miR-29b-3p or control, and after 48 h of transfection, total RNA was isolated using TRIzol reagent (Invitrogen). RNA was also isolated from xenograft tumors from both groups. cDNA was synthesized using miR-29b-3p or U6-specific primers with a TaqMan microRNA reverse transcription kit. Real-time PCR was performed for quantitation of gene expression using TaqMan Universal PCR master mix and 6-carboxyfluorescein (FAM)-MGB probes for miR-29b (assay ID: 000413) as per manufacture’s protocol (ThermoFisher Scientific, Waltham, MA, USA). U6 (assay ID: 001973) was used as an endogenous control. For Bim expression, cDNA was synthesized using a random hexamer with Superscript III reverse transcriptase. Real-time PCR was performed for quantitation of gene expression using SYBR green for Bim specific primers (IDT: Hs.PT.58.3264317). GAPDH was used as an endogenous control. The relative gene expression was analyzed by using the 2^−∆∆CT^ formula (ΔΔCT = ΔCT of the sample − ΔCT of the untreated control).

### 2.4. Cell Proliferation Assay

Trypan blue exclusion method was used to investigate cell proliferation in PC3 cells with control or mimic miR-29b. At 0, 24, 48 and 72 h of transfection, cells were harvested with trypsin, stained with trypan blue and percentage of live cells were counted using a hemocytometer. The cell numbers of control or transfected cells were compared with the number at 0 h. The experiment was performed in triplicate.

### 2.5. Cell Death Assay

PC3 cells were transfected with control or miR-29b mimic and were assayed for cell viability/death after 48 h of transfection using LIVE/DEAD Viability/Cytotoxicity Kit (Molecular Probes, CA, USA) as described before [17]. Cells were washed in PBS and exposed to 8 μM calcein-AM and 8 μM ethidium homodimer in PBS for 45 min at room temperature. Dye uptake was detected for fluorescein (for calcein in live cells) and Texas red (for ethidium homodimer in dead cells).

### 2.6. Transcriptomic Analysis Using a Human Cancer Pathway Finder Array

A total 1 µg high quality total RNA (RNA 260/280 ratio > 1.8) was reverse transcribed using first strand synthesis kit (Qiagen) and Human Cancer Pathway Finder RT^2^ profiler PCR Array (Qiagen) was performed to analyze the modulation of 84 cancer related genes according to the manufacturer’s instruction as described previously [18]. Array data were analyzed using web based software automatically performing all ^ΔΔ^Ct fold change calculations comparing experimental PC3 cells overexpressing mimic miR-29b-3p to control PC3 cells (http://pcrdataanalysis.sabiosciences.com/pcr/arrayanalysis.php).

### 2.7. Western Blot Analysis

Cell lysates were analyzed by SDS-PAGE and transferred onto 0.45 μm nitrocellulose membrane (Bio-Rad). Membranes were blocked using 5% low-fat dry milk in TBST and probed with specific antibodies to Bim (1:1000; Cell Signaling Technology), total PARP (1:1000; Cell Signaling Technology) and then HRP-conjugated anti anti-rabbit secondary antibodies (1:2000, Cell Signaling Technology). The blot was reprobed with β-Actin antibody (1:5000, Santacruz Biotechnology) to compare protein load in each lane. Densitometry analysis was done by using Image J software.

### 2.8. Immunofluorescence Analysis

PC3 cells were transfected with control or miR-29b mimic and were fixed with chilled methanol for 5 min at −20 °C. After blocking with 5% BSA for 1 h at room temperature, primary antibody cytochrome C (1:250, BD Biosciences) was added for overnight at 4 °C, followed by anti-mouse immunoglobulin conjugated to Alexa Fluor 647 (Molecular Probes) for 1 h at room temperature. Cells were counter stained with DAPI (4′,6′-diamidino-2-phenylindole) for nuclear staining (Molecular Probes). Two-channel optical images (red and blue) were collected using the sequential scanning mode of the Olympus FV1000 confocal system. Whenever necessary, the images were merged digitally to monitor co-localization in which two different colors produce a distinct color, whereas physically separate signals retain their individual colors.

### 2.9. Statistical Analysis

Results are expressed as the mean ± standard deviation (SD), and statistical analyses were performed using a two-tailed Student’s t test in Microsoft Excel. A *p*-value of <0.05 was considered statistically significant.

## 3. Results

### 3.1. miR-29b Inhibits Prostate Xenograft Tumor Growth

**Figure 1:**
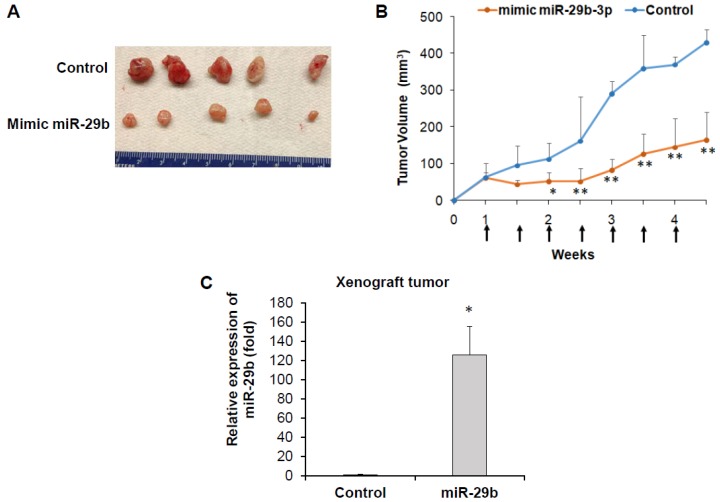
miR-29b regresses in-vivo prostate tumor growth. PC3 cells were injected subcutaneously into the flank of nude mice. When tumor volume reached ~70 mm^3^, mice were divided into two groups and 10 µg of miR-29b mimic or control oligoes were injected intratumorally at an interval of 4 days. (**A**) Representative tumors from control and experimental mice are presented. (**B**) Tumor volume was monitored twice weekly and presented as a mean. Small bar indicates standard error (*, *p* < 0.05, ** *p* < 0.01). Up arrows indicate treatment time points. (**C**) Relative expression of miR-29b in control and experimental tumors analyzed by qRT-PCR. U6 gene was used as internal control. Small bar indicates standard error (*, *p* < 0.05).

We examined whether mimic miR-29b treatment inhibits tumor growth in a PC3 xenograft mouse model. For this, we subcutaneously injected PC3 cells into hind flank of nude mice. When tumors were palpable (>70 mm^3^), mice were randomly divided into two groups, control and experimental groups. Control or mimic miR-29b was delivered by intratumor injection. We observed a significant reduction in tumor volume in mice receiving intratumoral injections of mimic miR-29b as compared to control mice (Figure 1A,B). At the end of the experiments, mice were sacrificed and tumors were removed for biochemical analysis. We did not observe any apparent toxicity. The body weight was similar in both groups and mice were macroscopically normal. miR-29b expression was examined in control mice and experimental mice. We observed significant up-regulation of miR-29b in experimental tumor compared to control tumors (Figure 1C). This result indicates a therapeutic potential of miR-29b against in vivo prostate tumor growth.

### 3.2. Overexpression of miR-29b Inhibits Prostate Cancer Cells Growth

**Figure 2:**
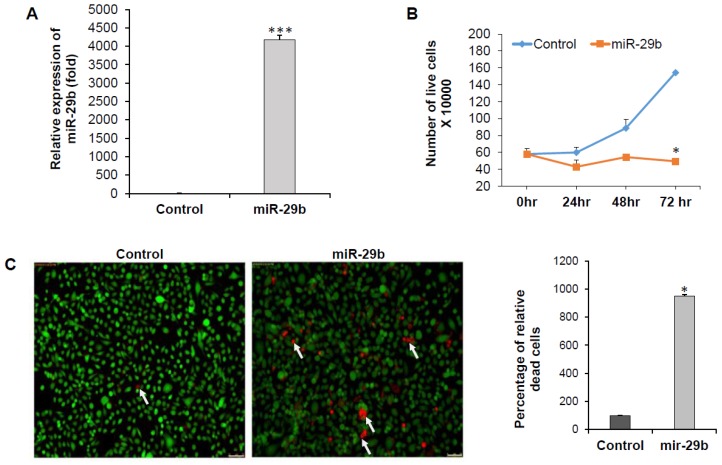
miR-29b inhibits prostate cancer cell growth. (**A**) PC3 cells were transfected with control or mimic miR-29b (50 nM). Expression of miR-29b was examined by qRT-PCR 48 h post-transfection. U6 gene was used as internal control. (**B**) PC3 cells were transfected with control or mimic miR-29b. At 0, 24, 48, and 72 h, cells were stained with trypan blue and number of live cells was counted using a hemocytometer. Data are presented as mean ± SD from three independent experiments. (**C**) Control or miR-29b transfected PC3 cells were stained with Calcein AM (green color for live cells) and ethidium homodimer-1 (red color for dead cells) dye to quantitate the live and dead cells by fluorescence microscopy. Magnification 10X and Scale bar 75 µm. Arrows indicate dead cells. Right panel shows quantitation of dead cells, calculated from five random fields. Small bar indicates standard error (* *p* < 0.05; *** *p* < 0.001).

To understand the role of miR-29b on in vitro prostate cancer cell line we overexpressed miR-29b mimic in PC3 cells. As expected, we also observed significant upregulation of miR-29b in the cells (Figure 2A). We examined proliferation status by staining with trypan blue at different time points. We observed reduction in cell proliferation upon overexpression of miR-29b in time dependent manner and significant change was seen at 72 h after transfection as compared to the control cells (Figure 2B). We observed a significant increase in the number of dead cells upon miR-29b overexpression as compared to control (Figure 2C).

### 3.3. Overexpression of miR-29b Induces Bim Expression in Prostate Cancer

**Figure 3:**
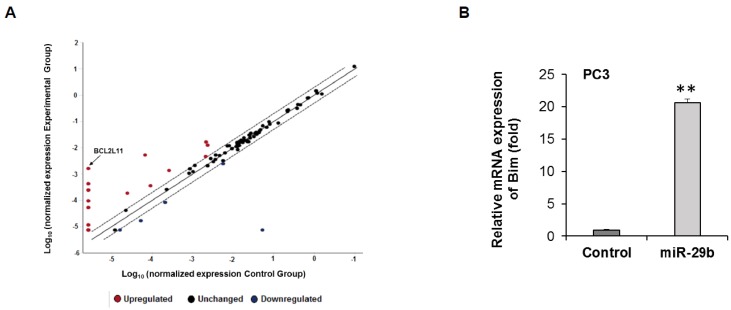
Transcriptomic analysis of miR-29b mimic transfected PC3 cells. (**A**) RNAs from control or miR-29b transfected **PC3 cells** were analyzed for pathway specific transcriptomic array using Human Cancer Pathway Finder RT^2^ profiler PCR Array (Qiagen). Relative fold change was analyzed using web-based software (Qiagen) using human β-Actin, β-2-microglobulin, glyceraldehyde-3-phosphate dehydrogenase, hypoxanthine phosphoribosyl transferase 1 and ribosomal protein, large, P0 genes as endogenous controls and presented graphically. (**B**) Total RNA was isolated from control or miR-29b transfected PC3 cells. Bim expression was measured by qRT-PCR and GAPDH was used as an internal control. The results are presented as mean ± SD from three independent experiments (**, *p* < 0.001).

**Figure 4:**
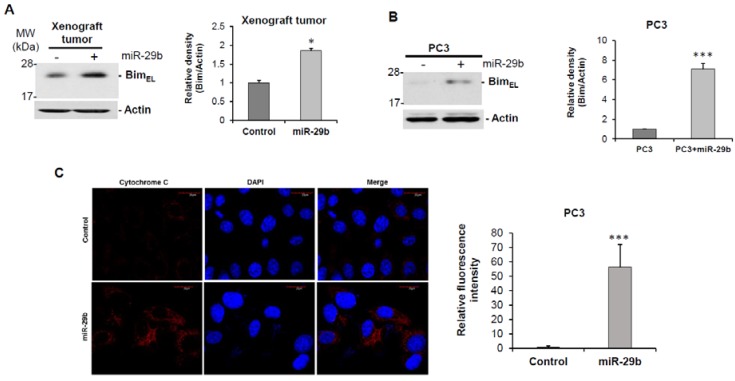

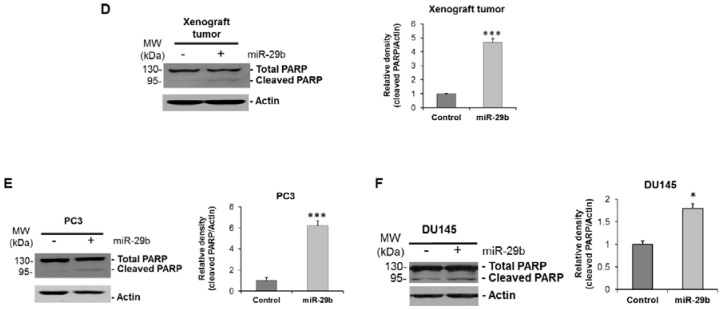
Overexpression of miR-29b induces Bim mediated apoptosis. Cell lysate from (**A**) xenograft tumors treated with mimic miR-29b and (**B**) PC3 cells were analyzed by Western blot using specific antibody to Bim. Extra-large transcript of Bim (Bim_EL_) was upregulated in mimic overexpressed samples. The membrane was reprobed with antibody to Actin as an internal control. Right panel shows quantitation of the data using Image J software. Densitometric scanning results are presented from three independent experiments. (**C**) PC3 cells were transfected with/without mimic miR-29b for 48 h and immune-stained with cytochrome C (red). DAPI (blue) was used for nuclearstaining. Representative confocal microscopic images showing increased cytoplasmic expression of cytochrome C in miR-29b transfected cells compared to control cells. Magnifications 60 X and scale bar 20 µm. Right panel shows quantitation of fluorescence intensity using ImageJ software using random five fields. Lysates of (**D**) xenograft tumors, (**E**) PC3 or (**F**) DU145 cells were subjected to Western blot analysis for PARP expression. Membrane was reprobed with Actin as internal control. Right panel shows quantitation of cleaved PARP using Image J software. Small bar indicates standard error (*, *p* < 0.05; *** *p* < 0.001).

To understand the molecular effect of miR-29b, we overexpressed miR-29b in PC3 cells, and performed a human cancer pathway finder profiling array. We analyzed 84 genes of cancer related pathways including angiogenesis, DNA damage, telomeres and telomerase, apoptosis, metabolism, cell cycle, epithelial to mesenchymal transition, hypoxia and senescence. We observed differential expression of these genes in mimic miR-29b overexpressed cells compared to control cells (Figure 3A). In the apoptosis pathway, pro-apoptotic gene BCL2L11 gene (Bim) was significantly upregulated in miR-29b overexpressed cells. To further verify the Bim expression, RNA was isolated from control or miR-29b transfected PC3 cells. Bim mRNA expression was measured by qRT-PCR and GAPDH was used as an internal control. Our result showed the higher expression of Bim in miR-29b transfected cells as compared to that of control cells (Figure 3B). Next, we examined the Bim protein expression in xenograft tumors treated with miR-29b and in mimic overexpressed PC3 cells. A significant upregulation of Bim protein was observed in both tumors and cell lines (Figure 4A,B).

### 3.4. miR-29b Induces Apoptotic Signaling in Prostate Cancer

Since we observed the elevated expression of pro-apoptotic molecule Bim we wanted to know whether miR-29b induces Bim mediated apoptosis. The Bcl2 –family protein Bim belongs to the BH3-only protein family known as initiators of apoptosis. It was evident that during cell death, Bim is constitutively incorporated in the outer mitochondrial membrane via a C-terminal transmembrane anchor from where it can induce cytochrome C release from mitochondria [19,20,21,22]. Immunofluorescent analysis revealed significant induction of cytosolic cytochrome C in miR-29b overexpressed PC3 cells as compared to the control cells (Figure 4C). Cytochrome C can initiate the activation of caspase cascade once it is accumulated in cytosol [23]. We also observed a significant induction of cleaved PARP in both xenograft tumors treated with miR-29b and mimic overexpressed PC3 cells as compared to controls (Figure 4D,E). A similar result was observed when miR-29b was overexpressed in DU145 cells as compared to that of control cells (Figure 4F). Thus, miR-29b inhibits tumor progression by inducing Bim and thereby inducing apoptosis signaling in prostate cancer.

## 4. Discussion

**Figure 5:**
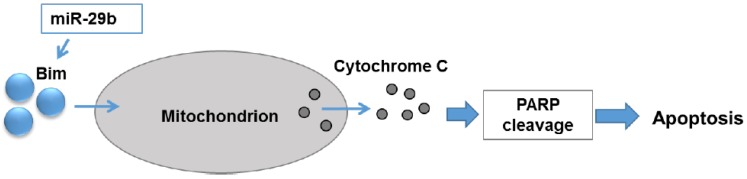
Schematic representation of role of miR-29b in induction of apoptosis in prostate cancer. miR-29b induces Bim expression resulting cytosolic accumulation of cytochrome C leading to PARP cleavage.

In this study, we have demonstrated that introduction of miR-29b mimic regresses prostate tumor growth in mice. Mechanistically, we have observed that miR-29b induced expression of pro-apoptotic molecule Bim, cytochrome C release and PARP cleavage in prostate cancer cells (Figure 5).

Studies in different in vivo mouse models demonstrated important role of miR-29b in development and normal physiological processes. Liver specific miR-29b knockout mouse models showed enhanced fibrosis and induction of carcinogenesis [24]. Lentivirus mediated the overexpression or intra-tumor injection of miR-29b regressed gastric tumor, acute myelogeneous leukemia, and multiple myeloma xenograft tumors [25,26,27]. We observed previously that the ectopic overexpression of miR-29b in PC3 cells failed to colonize in the lungs and liver of immunodeficient mice after intravenous injection [10]. In this study, we observed a potential therapeutic role of miR-29b upon intra-tumor delivery against prostate tumor development. We did not observe any side effect or loss of mice body weight and mice looked macroscopically normal following miR-29b treatment. Intra-tumoral injections of miRNA drugs enhance target specificity, efficacy, and minimize side effects [28,29]. Recently, miR-29 mimic MRG-201 has been tested in phase I clinical trials by injection into intact skin or adjacent to a short skin to evaluate its safety and tolerability in healthy volunteers (ClinicalTrials.gov: NCT02603224). Clinical trials that generally target a single molecule in biological pathways often fail to provide promising result. However, the use of miRNAs that efficiently regulate multiple cellular targets of different cellular pathways may have promising effects in cancer therapy.

We previously demonstrated that exogenous expression of miR-29b inhibited anti apoptotic molecule Mcl-1 and metastasis in prostate cancer [10,11]. In this study, using a cancer pathway specific transcriptomic array, we observed differential expression of several genes of apoptosis, cell cycle, proliferation and signaling pathways upon overexpression of miR-29b in PC3 cells. In agreement with our study, transcriptomic array of miR-29b overexpressed leukemia cells showed deregulation of similar pathways compared to the control cells [26]. We identified elevated expression of Bim in prostate cancer cells upon overexpression of miR-29b. AKT2, a validated target of miR-29b, suppresses transcription of pro-apoptotic gene Bim [15,16,21,30]. Pharmacological inhibitors that target Akt2 inducing Bim expression resulted in Bim mediated cellular apoptosis [30]. Similarly, tumor suppressor miRNA let-7 targets Akt pathway and induces Bim transcription [31]. Bim is a BH3-only BCL-2 family protein and effector of canonical mitochondrial apoptosis. Cancer cells suppress Bim expression which is associated with tumor promotion, metastasis, and drug resistance [21,22]. The overexpression of Bim inhibited tumor growth and drug resistance, and thus is gaining attention in chemotherapy [22]. Bim directly counteracts anti-apoptotic Bcl-xl in doxorubicin-induced apoptosis independently of p53 in prostate cancer cell lines PC3 and LNCaP and inhibition of Bim resulted in inhibition of caspase activation and apoptosis in these cell lines [32]. Several BH3 mimetics, e.g., ABT-737, ABT-263, obatoclax, AT-101 and A-1210477 were developed and are being used in current clinical trials [22]. Various chemotherapeutic agents like doxorubicin, paclitaxel, imatinib, dasatinib, nilotinib, gefitinib, erlotinib, and bortezomib regulate Bim expression and its signaling to the execution apoptotic cell death in breast cancer, colon cancer, lung cancer, and leukemia [21,32]. Overexpression of Bim can abrogate the function of many types of anti-apoptotic Bcl2 family proteins including Mcl-1 [21]. Induction of Bim triggers cytochrome C release from mitochondria to cytosol [21]. Overexpression of cytosolic cytochrome C induce apoptosis, and nanoparticle conjugated cytochrome C delivery into liver cancer cells either alone or in combination with doxorubicin, paclitaxel, oxaliplatin, vinblastine and vincristine could efficiently trigger cell death [33]. Cytosolic cytochrome C induces Caspase cleavage followed by PARP cleavage resulting in apoptosis [20,21,23]. Generally solid tumors lose the ability to undergo instantaneous and massive apoptosis, the so-called primary response that characterizes sensitive cells due to genetic mutations or alteration [34]. The induction of apoptosis is a common and required event for different classes of anticancer agents, and disruption of such mechanism can lead to broad drug resistance and sometimes non-specific side effects [34]. Thus, the interruption of such a mechanism and the induction of apoptosis may have an effect in the promotion of drug sensitivity and tumor regression. Since miR-29b expression was lowest in PC3 cells as compared to other prostate cancer cell lines tested previously [10], we included this cell line for the therapeutic efficacy of miR-29b. We will examine the phenotypic and molecular changes by overexpressing or depleting miR-29b in hormone sensitive prostate cancer cell lines in our future study. In summary, we demonstrated that the intratumor delivery of mimic miR-29b inhibits prostate tumor growth, in part through the Bim-mediated apoptosis pathway, suggesting its therapeutic potential in future clinical studies.

## Abbreviation

miRNA	microRNA
miR-29b	microRNA 29b- 3p
PARP	Poly [ADP-ribose] polymerase 1
Bim	Bcl2-interacting mediator of cell death

## References

[1] Siegel R.L., Miller K.D., Jemal A. (2019). Cancer statistics, 2019. CA Cancer J. Clin..

[2] Vanacore D., Boccellino M., Rossetti S., Cavaliere C., D’Aniello C., Di Franco R., Romano F.J., Montanari M., Mantia E.L., Piscitelli R. (2017). Micrornas in prostate cancer: An overview. Oncotarget.

[3] Xie M., Ma L., Xu T., Pan Y., Wang Q., Wei Y., Shu Y. (2018). Potential Regulatory Roles of MicroRNAs and Long Noncoding RNAs in Anticancer Therapies. Mol. Ther. Nucleic Acids.

[4] Chan J.J., Tay Y. (2018). Noncoding RNA: RNA Regulatory Networks in Cancer. Int. J. Mol. Sci..

[5] Rupaimoole R., Calin G.A., Lopez-Berestein G., Sood A.K. (2016). miRNA Deregulation in Cancer Cells and the Tumor Microenvironment. Cancer Discov..

[6] Hanna J., Hossain G.S., Kocerha J. (2019). The Potential for microRNA Therapeutics and Clinical Research. Front. Genet..

[7] Si W., Shen J., Zheng H., Fan W. (2019). The role and mechanisms of action of microRNAs in cancer drug resistance. Clin. Epigenet..

[8] Tong A.W., Fulgham P., Jay C., Chen P., Khalil I., Liu S., Senzer N., Eklund A.C., Han J., Nemunaitis J. (2009). MicroRNA profile analysis of human prostate cancers. Cancer Gene Ther..

[9] Pashaei E., Pashaei E., Ahmady M., Ozen M., Aydin N. (2017). Meta-analysis of miRNA expression profiles for prostate cancer recurrence following radical prostatectomy. PLoS ONE.

[10] Ru P., Steele R., Newhall P., Phillips N.J., Toth K., Ray R.B. (2012). miRNA-29b suppresses prostate cancer metastasis by regulating epithelial-mesenchymal transition signaling. Mol. Cancer Ther..

[11] Steele R., Mott J.L., Ray R.B. (2010). MBP-1 upregulates miR-29b that represses Mcl-1, collagens, and matrix-metalloproteinase-2 in prostate cancer cells. Genes Cancer.

[12] Zhu C., Hou X., Zhu J., Jiang C., Wei W. (2018). Expression of miR-30c and miR-29b in prostate cancer and its diagnostic significance. Oncol. Lett..

[13] Mott J.L., Kurita S., Cazanave S.C., Bronk S.F., Werneburg N.W., Fernandez-Zapico M.E. (2010). Transcriptional suppression of mir-29b-1/mir-29a promoter by c-Myc, hedgehog, and NF-kappaB. J. Cell. Biochem..

[14] Zhang X., Zhao X., Fiskus W., Lin J., Lwin T., Rao R., Zhang Y., Chan J.C., Fu K., Marquez V.E. (2012). Coordinated silencing of MYC-mediated miR-29 by HDAC3 and EZH2 as a therapeutic target of histone modification in aggressive B-Cell lymphomas. Cancer Cell.

[15] Yan B., Guo Q., Fu F.J., Wang Z., Yin Z., Wei Y.B., Yang J.R. (2015). The role of miR-29b in cancer: Regulation, function, and signaling. OncoTargets Ther..

[16] Kwon J.J., Factora T.D., Dey S., Kota J. (2019). A Systematic Review of miR-29 in Cancer. Mol. Ther. Oncolytics.

[17] Shrivastava S., Raychoudhuri A., Steele R., Ray R., Ray R.B. (2011). Knockdown of autophagy enhances the innate immune response in hepatitis C virus-infected hepatocytes. Hepatology.

[18] Rajamoorthi A., Shrivastava S., Steele R., Nerurkar P., Gonzalez J.G., Crawford S., Varvares M., Ray R.B. (2013). Bitter melon reduces head and neck squamous cell carcinoma growth by targeting c-Met signaling. PLoS ONE.

[19] Frank D.O., Dengjel J., Wilfling F., Kozjak-Pavlovic V., Hacker G., Weber A. (2015). The pro-apoptotic BH3-only protein Bim interacts with components of the translocase of the outer mitochondrial membrane (TOM). PLoS ONE.

[20] Lohmann C., Muschaweckh A., Kirschnek S., Jennen L., Wagner H., Hacker G. (2009). Induction of tumor cell apoptosis or necrosis by conditional expression of cell death proteins: Analysis of cell death pathways and in vitro immune stimulatory potential. J. Immunol..

[21] Akiyama T., Dass C.R., Choong P.F. (2009). Bim-targeted cancer therapy: A link between drug action and underlying molecular changes. Mol. Cancer Ther..

[22] Shukla S., Saxena S., Singh B.K., Kakkar P. (2017). BH3-only protein BIM: An emerging target in chemotherapy. Eur. J. Cell Biol..

[23] Cai J., Yang J., Jones D.P. (1998). Mitochondrial control of apoptosis: The role of cytochrome c. Biochim. Biophys. Acta.

[24] Kogure T., Costinean S., Yan I., Braconi C., Croce C., Patel T. (2012). Hepatic miR-29ab1 expression modulates chronic hepatic injury. J. Cell. Mol. Med..

[25] Wang T., Hou J., Jian S., Luo Q., Wei J., Li Z., Wang X., Bai P., Duan B., Xing J. (2018). miR-29b negatively regulates MMP2 to impact gastric cancer development by suppress gastric cancer cell migration and tumor growth. J. Cancer.

[26] Garzon R., Heaphy C.E., Havelange V., Fabbri M., Volinia S., Tsao T., Zanesi N., Kornblau S.M., Marcucci G., Calin G.A. (2009). MicroRNA 29b functions in acute myeloid leukemia. Blood.

[27] Amodio N., Leotta M., Bellizzi D., Di Martino M.T., D’Aquila P., Lionetti M., Fabiani F., Leone E., Gullà A.M., Passarino G. (2012). DNA-demethylating and anti-tumor activity of synthetic miR-29b mimics in multiple myeloma. Oncotarget.

[28] Mercatelli N., Coppola V., Bonci D., Miele F., Costantini A., Guadagnoli M., Bonanno E., Muto G., Frajese G.V., Maria R.D. (2008). The inhibition of the highly expressed miR-221 and miR-222 impairs the growth of prostate carcinoma xenografts in mice. PLoS ONE.

[29] Chen Y., Gao D.Y., Huang L. (2015). In vivo delivery of miRNAs for cancer therapy: Challenges and strategies. Adv. Drug Deliv. Rev..

[30] Xie M., Yang A., Ma J., Wu M., Xu H., Wu K., Jin Y., Xie Y. (2019). Akt2 mediates glucocorticoid resistance in lymphoid malignancies through FoxO3a/Bim axis and serves as a direct target for resistance reversal. Cell Death Dis..

[31] Lin X., Shen J., Dan P., He X., Xu C., Chen X., Tanyi J.L., Montone K., Fan Y., Huang Q. (2018). RNA-binding protein LIN28B inhibits apoptosis through regulation of the AKT2/FOXO3A/BIM axis in ovarian cancer cells. Signal Transduct. Target. Ther..

[32] Yang M.C., Lin R.W., Huang S.B., Huang S.Y., Chen W.J., Wang S., Hong Y.R., Wang C. (2016). Bim directly antagonizes Bcl-xl in doxorubicin-induced prostate cancer cell apoptosis independently of p53. Cell Cycle.

[33] Al-Shakarchi W., Alsuraifi A., Abed M., Abdullah M., Richardson A., Curtis A., Hoskins C. (2018). Combined Effect of Anticancer Agents and Cytochrome C Decorated Hybrid Nanoparticles for Liver Cancer Therapy. Pharmaceutics.

[34] Ferreira C.G., Epping M., Kruyt F.A., Giaccone G. (2002). Apoptosis: Target of cancer therapy. Clin. Cancer Res. Off. J. Am. Assoc. Cancer Res..

